# Ancient acquisition of “alginate utilization loci” by human gut microbiota

**DOI:** 10.1038/s41598-018-26104-1

**Published:** 2018-05-23

**Authors:** Sophie Mathieu, Mélanie Touvrey-Loiodice, Laurent Poulet, Sophie Drouillard, Renaud Vincentelli, Bernard Henrissat, Gudmund Skjåk-Bræk, William Helbert

**Affiliations:** 1 CERMAV, CNRS and Grenoble Alpes Université, BP53, 38000 Grenoble Cedex 9, France; 2 Centre National de la Recherche Scientifique (CNRS), UMR7257, Université Aix-Marseille, Marseille, 13288 France; 3 INRA, USC 1408 AFMB, 13288 Marseille, France; 4 Department of Biological Sciences, King Abdulaziz University, Jeddah, Saudi Arabia; 5 Department of Biotechnology, Norwegian University of Science and Technology, NTNU Sem Sælands vei 6-8, 7491 Trondheim, Norway

## Abstract

In bacteria from the phylum *Bacteroidetes*, the genes coding for enzymes involved in polysaccharide degradation are often colocalized and coregulated in so-called “polysaccharide utilization loci” (PULs). PULs dedicated to the degradation of marine polysaccharides (e.g. laminaran, ulvan, alginate and porphyran) have been characterized in marine bacteria. Interestingly, the gut microbiome of Japanese individuals acquired, by lateral transfer from marine bacteria, the genes involved in the breakdown of porphyran, the cell wall polysaccharide of the red seaweed used in maki. Sequence similarity analyses predict that the human gut microbiome also encodes enzymes for the degradation of alginate, the main cell wall polysaccharide of brown algae. We undertook the functional characterization of diverse polysaccharide lyases from family PL17, frequently found in marine bacteria as well as those of human gut bacteria. We demonstrate here that this family is polyspecific. Our phylogenetic analysis of family PL17 reveals that all alginate lyases, which have all the same specificity and mode of action, cluster together in a very distinct subfamily. The alginate lyases found in human gut bacteria group together in a single clade which is rooted deeply in the PL17 tree. These enzymes were found in PULs containing PL6 enzymes, which also clustered together in the phylogenetic tree of PL6. Together, biochemical and bioinformatics analyses suggest that acquisition of this system appears ancient and, because only traces of two successful transfers were detected upon inspection of PL6 and PL17 families, the pace of acquisition of marine polysaccharide degradation system is probably very slow.

## Introduction

The high complexity of polysaccharides arises from the stereochemical diversity of their constitutive monosaccharide residues and from the many ways to assemble these residues through glycosidic bonds. Primarily produced by photosynthesis, these macromolecules represent the most abundant biomass and carbon source for land and marine heterotrophic organisms. The biodegradation of polysaccharides requires large arrays of enzymes that can specifically cleave each of the constitutive glycosidic bonds, either by hydrolysis (glycoside hydrolases, GH), β-elimination (polysaccharide lyases, PL) or oxidation (lytic polysaccharide mono-oxygenases, LPMO). Thus, the complexity of the polysaccharide substrates is accompanied by a wide diversity of polysaccharide-degrading enzymes. The International Union of Biochemistry and Molecular Biology lists more than 200 EC numbers that correspond to different GH substrate specificities (http://www.enzyme-database.org)^1^ and the carbohydrate-active enzymes database (CAZy database, http://www.cazy.org/)^2^ features 145 GH, 27 PL and 13 LPMO sequence-based families, and these numbers are still growing, *e*.*g*. the recent addition of seven new GH families^3^.

Members of the individual sequence-based families of carbohydrate-active enzymes listed in the CAZy database share the same folds and the same catalytic amino acids^2^. In the case of GHs, the orientation of the cleaved glycosidic bond and the stereochemical outcome of the reaction (e.g. retaining or inverting the anomeric configuration at the site of cleavage) are essentially conserved in each family^4,5^. However, many sequence-based families are polyspecific, *i*.*e*. group enzymes of different substrate specificity. For several large families of GHs, subfamilies have been shown to improve correlation with substrate specificity and also to identify clades that contain enzymes without any experimentally demonstrated function^6–9^. Similarly, several families of PLs have been discovered to be polyspecific and most of them have also been divided into subfamilies^10^. In contrast to the GH families however, PL families contain fewer members and the conservation of function within subfamilies is probably less robust^11^, making functional predictions from sequence data alone less reliable.

In bacteria belonging to phylum *Bacteroidetes*, genes involved in the degradation of a given polysaccharide are often clustered on the genome and coregulated. These gene clusters have been termed “polysaccharide utilization loci” (PULs)^12,13^. This colocalization offers an additional lever for the prediction of the function of other polysaccharide-degrading enzymes when one or several members of a PUL have been biochemically characterized. Over 26,000 predicted PULs are listed in the CAZy database (http://www.cazy.org/PULDB/) based on the analysis of 820 bacteria from the human gut microbiote and from the environment^14,15^. Analysis of the marine bacterial genomes or metagenomes suggests a large diversity of PULs for the breakdown of marine polysaccharides^16–22^. However, the precise function (specificity) of the PULs can only be hypothesized based on sequence similarity of protein sequences found in the PUL with characterized, but distantly related, carbohydrate-active enzymes. Hypotheses on the specificity of marine PULs are usually formulated by confronting the putative function of genes with the available literature on the composition or structure of marine polysaccharides. The function of several PULs involved in the degradation of marine polysaccharides has been demonstrated by transcriptomics, proteomics or enzymology methods. There are marine PULs that target laminaran and alginate^18^ (the storage and cell wall polysaccharides of brown algae, respectively), ulvan^22^ (the cell wall polysaccharide of the green algae *Ulva* and *Enteromorpha* sp.), and porphyran^23^ (the cell-wall polysaccharide of the red alga *Porphyra* sp.).

Porphyran-degrading enzymes (porphyranases) have reportedly been horizontally transferred from a marine *Bacteroidetes* to *Bacteroidetes* harbored in the Japanese gut microbiota^23^. Acquisition of the porphyran-degrading enzymes has been attributed to the algae-rich diet common in Japan, suggesting that contaminated raw food may have been the vector of the transferred genes. Porphyranases are glycoside hydrolases that belong to the polyspecific GH16 family, which also contains agarases and carrageenases and other enzymes for the degradation of red algal polysaccharides. Phylogenetic analyses of family GH16 show that the porphyranase clade groups with both marine and gut *Bacteroidetes* and shares a common ancestor with agarases and carrageenases^23^. These results highlight the plasticity of the gut microbiome and its ability to adapt to diet. However, the frequency of acquisition of a novel gene introduced by diet and the mechanisms of adaptation to diet remain obscure.

The possible occurrence of alginate lyases and carrageenases in the human gut microbiome has been suggested based on sequence similarity of proteins encoded by gut bacteria to alginate lyases^24^ and on the fact that fecal bacteria can degrade carrageenan^25^. However, the functional characterization of the corresponding proteins has not been performed, leaving porphyran as the only experimentally demonstrated example of a marine polysaccharide degraded by enzymes found in the human gut microbiome. Here, we conducted a functional characterization of alginate lyases from family PL17 to establish that human gut bacteria do encode an alginate-degradation system and, combined with phylogenetic analyses, we discuss the putative evolutionary steps that led to the transfer of marine polysaccharide degradation systems to the human gut microbiome.

## Results

### Selection and production of recombinant PL17 enzymes

**Figure 1:**
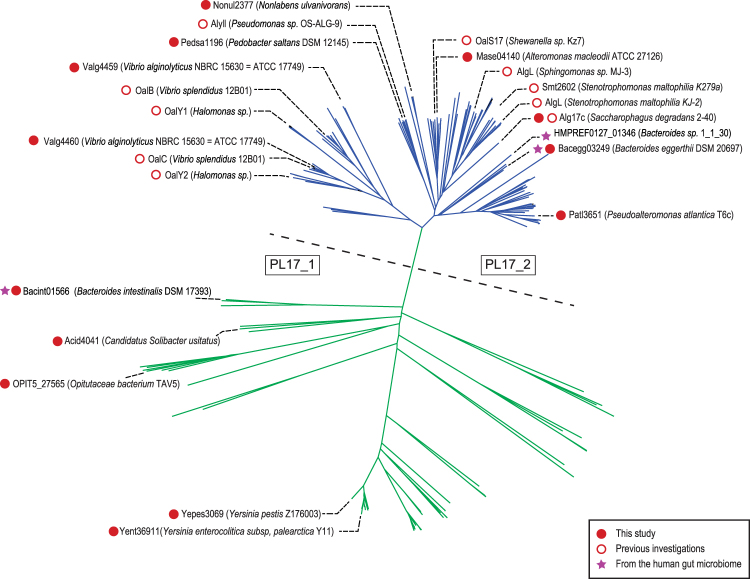
Phylogenetic tree of the PL17 family. The tree can be divided into two main clades, separated here by a dashed line. In the clade above the dashed line, shown in blue, all the characterized enzymes are exo-mannuronan lyases. The characterized enzymes below the dashed line, shown in green, include hyaluronic and poly-glucuronic acid lyases.

The phylogenetic tree computed from the alignment of the catalytic modules of 272 PL17 sequences shows a clear division into two main clades (Fig. 1). One clade contains all the biochemically characterized enzymes already described in the family, all being alginate lyases (shown in blue in Fig. 1). This clade corresponds to subfamily PL17_2^10^. The other clade (shown in green in Fig. 1) forms a less homogenous group with lineages that root deeply, reflecting high sequence divergence. This clade groups the members of subfamily PL17_1 along with sequences not assigned to any subfamily. To the best of our knowledge, no enzymes from this highly divergent clade have been characterized experimentally and thus the specificity of the corresponding proteins cannot be reliably predicted. Subfamily classification improves substrate specificity predictions when subfamilies contain many characterized and isofunctional members; however, predictive power decreases with the size of dataset and, as illustrated in the PL6 family, when several substrate specificities and modes of action (endo- or exo-lyases) are found in the same subfamily^11^. The shape of the phylogenetic tree of family PL17, the small number of published protein sequences and the absence of any characterized members in a distantly related clade suggest that there may be other specificities or modes of action. To explore the functional diversity in family PL17, we selected 13 target proteins spread across the phylogenetic tree for production and enzymatic characterization.

All the targeted proteins fused to a His_6_-tag were successfully overexpressed in a soluble manner in *E*. *coli* BL21(DE3) grown overnight at 20 °C. The proteins were purified by affinity and size-exclusion chromatography. The proteins were analyzed by SDS-PAGE and displayed molecular weights ranging from 71.2 to 84.7 kDa, as expected. As reported for the *Saccharophagus degradans* alginate lyase^26^, size-exclusion chromatography showed that several proteins such as Valg4460, Pedsa1196 and Mase04140 form dimers, but dimerization was not detected consistently in our experimental conditions.

### Functional screening of PL17 family members

Alginates extracted from brown algae are composed of d-mannuronic acid (M) and l-guluronic acid (G) residues distributed in blocks or alternately along the polysaccharide chain. Composition and distribution of the uronic residues vary greatly according to the algal species^27^. Isolation of the blockwise fractions of poly-M and poly-G substrates can be obtained by acid hydrolysis followed by selective precipitation experiments^28^, but highly pure material is difficult to prepare. Therefore, to obtain an accurate description of substrate specificities, we used a poly-M substrate with a regular structure biosynthesized by genetically engineered *Pseudomonas fluorescens* to avoid the presence of l-guluronic acid residues in the composition^29^. Regular poly-MG chains were obtained after enzymatic epimerization, which led to a substrate devoid of G-block contamination^30^. Substrates with G-blocks were obtained by acid hydrolysis of alginate, but some traces of M-residues were still present^28^.

**Figure 2:**
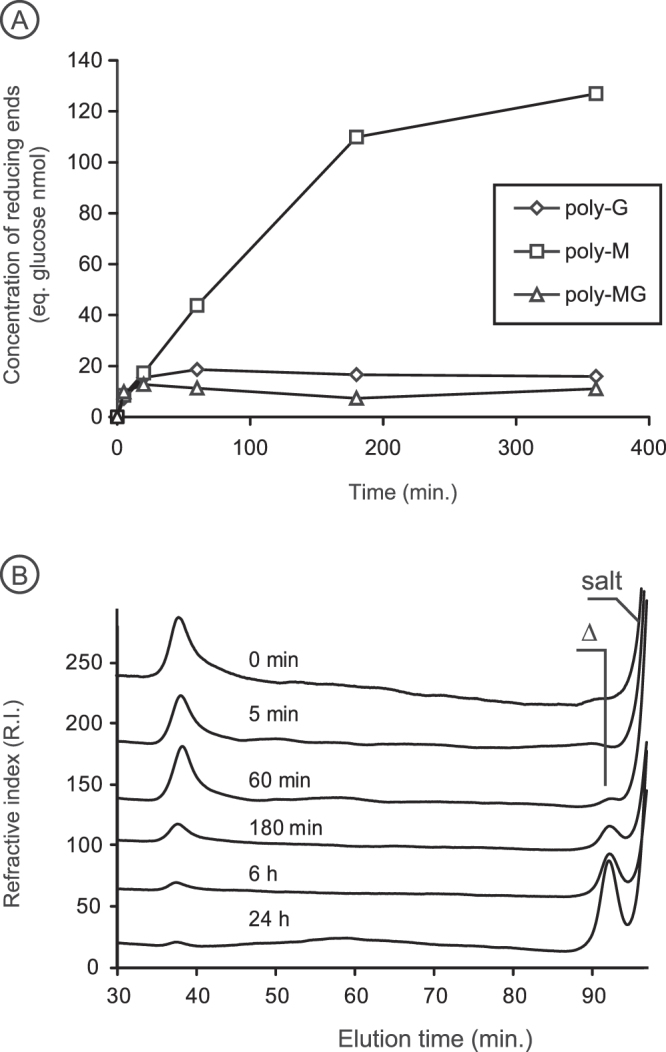
Characterization of the exo-acting mannuronan (M) lyase Nonul2377. (**A**) Representative degradation kinetic revealing the poly-M substrate specificity. Incubation on standard alginate substrates was conducted, at least, with two independent production of enzymes. (**B**) The exolytic mode of action of the enzymes was visualized using size-exclusion chromatography. Δ: 4-deoxy-L-erythro-5-hexoseulose uronic acid.

The eight proteins selected from the PL17_2 clade all displayed alginate lyase activity (Table 1), as reported for other members of this clade based on digestion experiments conducted with either raw alginate substrates or on isolated poly-M or poly-G block fractions prepared by chemical treatment^26,31–35^. The substrate specificity of the enzymes was determined using at least two independent production of enzymes by measuring the velocity of degradation using poly-M, poly-G and poly-MG substrates (Fig. 2A). The degradation kinetics revealed that the preferred substrate was always poly-M chains. For Pedsa1196 (*Pedobacter saltans*) and Mase04140 (*Alteromonas macleodii*), the enzymes also showed weak activity on the poly-MG substrate (Table 1). The degradation of poly-M chains monitored by size-exclusion chromatography revealed that the alginate lyases produced a single unsaturated monosaccharide end product (4-deoxy-L-erythro-5-hexoseulose uronic acid) (Fig. 2B). No product of intermediate size was produced, demonstrating that the alginate lyases of the clade PL17_2 are exo-polymannuronan lyases.

**Table 1:** Substrate specificity and mode of action.

Locus	Strain	Preferred substrate	End products (Mode of action)	Other substrates	End products (Mode of action)
**Subfamily 1**					
Acid4041	“*Candidatus* Solibacter usitatus”	Hyaluronan	DP2, DP4 (Endo)		
*OPIT5_27565*	*Opitutaceae bacterium* TAV5	Poly-glucuronic acid			
Bacint01566	*Bacteroides intestinalis*	n.d.			
Ypes3069	*Yersinia pestis* Z176003	n.d.			
Yent36911	*Yersinia enterocolitica* subsp. *palearctica* Y11	n.d.			
**Subfamily 2**					
Pedsa1196	*Pedobacter saltans*	Poly-M	Δ^**^ (Exo)	Poly-MG	Δ (Exo)
Mase04140	*Alteromonas macleodii*	Poly-M	Δ (Exo)	Poly-MG/Poly-G	Δ (Exo)
Alg17c	*Saccharophagus degradans* 2–40	Poly-M	Δ (Exo)		
Valg4459	*Vibrio alginolyticus*	n.d.			
Valg4460	*Vibrio alginolyticus*	n.d.			
Bacegg03249	*Bacteroides eggerthii*	Poly-M	Δ (Exo)		
Patl3651	*Pseudoalteromonas atlantica* T6c	Poly-M	Δ (Exo)		
Nonul2377	*Nonlabens ulvanivorans*	Poly-M	Δ (Exo)		
AlyII^31^	*Pseudomonas* sp. OS-ALG-9	Poly-M^*^	n.d.		
AlgL^32^	*Sphingomonas* sp. MJ-3	Poly-M^*^	n.d.	Poly-MG/Poly-G	n.d.
OalY1, OalY2^33^	*Halomonas* sp.	Poly-M^*^	Δ (Exo)		
Alg17c^26^	*Saccharophagus degradans* 2–40	Oligo-alginates	Δ (Exo)		
AlgL^34^	*Stenotrophomas maltophilia* KJ-2	Poly-M^*^, Oligo-alginates	n.d.		
Smlt2602^35^	*Stenotrophomonas maltophilia* K279a	Poly-M^*^	Δ (Exo)		
OalB^51^	*Vibrio splendidus* 12B01	Poly-MG^*^	n.d.		
OalC^51^	*Vibrio splendidus* 12B01	Poly-M	n.d.		
OalS17^52^	*Shewanella* sp.Kz7	Poly-M	n.d.		

^*^The substrates used to characterize the enzyme specificities were alginate or enriched poly-M or poly-G fractions obtained by acid hydrolysis.

^**^Δ: 4-deoxy-L-erythro-5-hexoseulose uronic acid

The table summarizes the substrate specificities, end products and mode of action (endo-, exo-) of the polysaccharide lyases investigated. The alginate lyases described in the literature are also reported in the table (shaded in gray).

The five proteins selected from the other clade were all inactive on alginates extracted from algae or on alginates with regular structures (poly-M, poly-G and poly-MG). They were thus assayed with a collection of oligo- and polysaccharides containing uronic residues in their structure. No degradation of oligo- or polysaccharides used in this work was observed for the proteins Bacint01566 (*Bacteroides intestinalis*, ZP_03014006), Ypes3069 (*Yersinia pestis*, Z176003) and Yent36911 (*Yersinia enterocolitica* subsp. *palearctica* Y11, CBY28839). However, Acid4041 (“*Candidatus* Solibacter usitatus”, ABJ85006) degraded hyaluronic acid according to an endolytic mode of action giving di- and tetrasaccharide end products. Other substrates with β-linked glucuronic acid residues in their structures (e.g. poly-glucuronic acid, xanthan) were not degraded (Fig. S1A). OPIT5_27565 (*Opitutaceae bacterium* TAV5, WP_009508481) was found to be active on poly-β(1,4)-D-glucuronic acid. This enzyme had an endo- mode of action and the smallest end products were di- and trisaccharides (Fig. S1B). All the screening results, substrate specificities and modes of action are summarized in Table 1.

## Discussion

### Polyspecificity of family PL17

Alginate lyases were all found in the same clade corresponding to the PL17_2 subfamily and they all shared the same exo-M-lyase mode of action. Most of these enzymes are of marine origin, consistent with the degradation of alginate from marine brown algae. The specificity and the mode of action of the alginate lyases were carefully determined using alginate substrates with known, controlled structures (poly-M, poly-G or poly-MG chains). The results were in agreement with the previous enzymology and crystallography studies carried out on other recombinant enzymes^26,31–35^. One report indicates that the expression of the marine strain *Gramella forsetti* PL17 enzyme is upregulated when alginate is added to the culture medium^18^, supporting substrate specificity towards alginate. Similarly, the transcription of *Zobellia galactinovorans* PL17 has been induced in the presence of alginate^24^. Therefore, the clade defining the PL17_2 subfamily appears monospecific in light of all data recorded to date.

The results obtained with the PL17 members outside of the first clade were more contrasted. We were not able to identify the substrate for three enzymes (Bacint01566, Ypes3069, Yent36911), but we identified the function of two enzymes: namely, hyaluronan lyase for Acid 4041 and glucuronan lyase for OPIT5_27565. These two activities have never been reported before in family PL17. The hyaluronan and β-1,4-glucuronan substrates share structural similarities, especially the β-linked glucuronic acid residue which undergoes enzymatic cleavage by β-elimination. Both enzymes had an endo-acting mode of action, in contrast to the exo-M lyases. Thus, differences in substrate specificity and mode of action accompany the segregation of the sequences into two clades on the PL17 phylogenetic tree. Our characterization of hyaluronan and glucuronan lyases in family PL17 also indicates that the previously reported functional data were not sufficient to accurately predict the function of PL17 members based on sequence similarity, and that the poly-specificity of this family has clearly been underestimated. In addition, because several enzymes in our study did not show activity on any of the substrates we used, it is also likely that there are other specificities. In any case, caution should be taken when attempting to assign a function to uncharacterized members that are only distantly related to alginate, glucuronan and hyaluronan lyases.

### Alginate utilization loci in human gut microbiota

**Figure 3:**
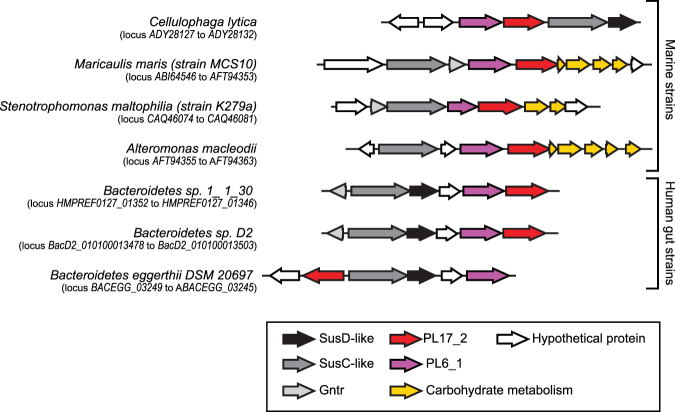
Alginate utilization loci in the human gut microbiome containing PL17 alginate lyase. The organization of these PULs were compared with marine alginate lyase loci.

The genes coding for enzymes involved in the recognition, internalization and degradation of oligomers and alginate polymers as well as enzymes associated with the metabolic pathways of the end products are colocalized in PULs in the genome of several marine *Bacteroidetes* strains^18,24^. In the PULDB database (www.cazy.org/PULDB/), which lists the PULs predicted in a large number of *Bacteroidetes*, we observed that all PULs of the human gut microbiote containing PL17 alginate lyases also contain alginate lyases from the PL6_1 subfamily (Fig. 3). Recent functional investigations have shown that the members of the PL6_1 subfamily are endo-alginate lyases^11^. Therefore, the exo-acting PL17_2 enzymes likely cleave the oligo-alginates produced by the PL6_1 endo-acting lyases. The conservation of colocalized gene pairs coding for PL17_2 and PL6_1 alginate lyases probably reflects a conserved alginate-degradation system.

In addition to the conserved pairs of PL6_1 and PL17_2 genes, the physical organization of the genes in several PULs appears well conserved in several bacteria regardless of their origin – be it marine or human gut (Fig. 3). Using a homology-based approach, Thomas and co-workers^24^ hypothesized that the alginate degradation system has been laterally transferred from marine to human gut *Bacteroidetes*. The detailed biochemical characterization of families PL6 and PL17 now paves the way to a more robust functional prediction for members of these families. In particular, our experimental data can discriminate true “alginate utilization loci” from other PULs and especially exclude those containing PL17 proteins that do not cluster with alginate lyases in the phylogenetic tree. Interestingly, the exo-M-lyase activity that we found for *Bacteroides eggerthii* (Baceg_03249) demonstrates the occurrence of an active alginate lyase in the human gut microbiome.

### Ancient acquisition of an alginate utilization locus by the human gut microbiome

The phylogenetic tree of family PL17 shows that human gut alginate lyases root deeply in the tree (Fig. 1). The mode of action *B*. *eggerthii* (Baceg_03249) is similar to all other alginate lyases and its genomic environment (*i*.*e*. PUL organization) is well preserved, suggesting that the divergence of protein sequences cannot be attributed to a major functional shift. The deep root has one of two explanations. Either it reflects the recent transfer of a PL17 gene belonging to an environmental strain, not present in sequence databanks, that has evolved independently from the other lineages, or it is the ancient acquisition of the PL17 gene by the human gut microbiome followed by independent evolution.

To test these hypotheses, we compared the G + C ratio of the alginate lyases in the PULs with that in the corresponding full genome. The G + C ratios of the genomes of the human gut reference strains (*Bacteroidetes* sp. 1_1_30, *Bacteroides* sp. D2, *Bacteroides* sp. 2_2_4, *Bacteroides* sp. 3_1_23, *Bacteroides clarus* YIT 12056) were very similar to that of the alginate utilization loci, suggesting old acquisition of the alginate lyases by these organisms. In contrast with these five reference strains, the alginate utilizing loci of *B*. *eggerthii* DSM 20697 has a different gene organization and had a G + C ratio very different from that of the full genome suggesting it has been the subject of lateral gene transfer^36^. Because all the PL17 alginate lyases of the human gut clustered in the same clade of the phylogenetic tree, it is thus likely that – after an ancient horizontal transfer – the human microbiome alginate lyase genes evolved under selection pressures that differ from those experienced by the original donor. The transfer of PL17 alginate lyase genes between bacteria of the human gut microbiota probably occurred more than once, explaining the distinctive distribution of genes from several strains and the probable recent acquisition of *B*. *eggerthii* DSM 20697 alginate lyase from a different donor.

Due to the colocalization of the PL6 and PL17 alginate lyases in PULs, the acquisition of PL6 alginate lyases is likely to have followed a very similar scenario. We built a phylogenetic tree of family PL6 including all PL6 alginate lyases predicted in the known strains of the human gut microbiota (Fig. S2). The gut alginate lyases grouped into two distinct branches that rooted deep in the tree. One branch groups all the PL6_1 genes that colocalize with PL17_2 alginate lyases, indicating a common ancestor for these PL6_1 enzymes. The second clade contains PL6 alginate lyase genes that were localized in a PUL composed of only PL6 alginate lyases. Altogether, this suggests that PL6 alginate lyases were probably acquired by the gut microbiota twice, from two donor strains that possessed PL6 alginate lyases in different genomic environments.

The phylogenetic analyses of the PL17_2 and PL6_1 alginate lyases colocalized in the same PUL suggest a single ancient acquisition of this PUL by a member of the human gut microbiota. Comparison of the phylogenetic trees is not a direct proof of the co-acquisition of PL17_2/PL6_1 alginate lyases; however, due to their complementary mode of action and their co-occurrence in many marine strains, their co-transfer from marine to gut *Bacteroidetes* appears highly likely. The retention of the PL17/PL6 gene pairs in the genomes of human gut bacteria for a long period of time supposes that the functional enzymes were expressed and conferred an advantage to the host strain. Therefore, it is reasonable to assume that all the elements of the alginate utilization locus, including the machinery for the detection of the alginate substrate and the activation of alginate lyase expression, were all transferred *en bloc* from a marine to a human gut *Bacteroidetes*.

### Food: a vehicle of genes and a selective pressure on transferred genes

Lateral transfer between a marine strain and human gut *Bacteroidetes* was first demonstrated in a gene coding for the porphyranase involved in the degradation of the red algal cell wall polysaccharides found in sushi roll (maki) ingredients^23^. It was then suggested that genes were transferred to the human gut from marine *Bacteroidetes* associated with algae eaten as food. Analyses of the phylogenetic trees of PL17, PL6 and GH16 (which includes agarases and porphyranases) suggest that, despite the frequent consumption of algae over many generations, the transfer of genes coding for marine polysaccharide-degrading enzymes is quite rare and ancient. Genomic data available to date are a snapshot of the genetic capital of the microbiome. Possible temporary acquisition of other alginate degradation systems – now lost – may have occurred. The bioinformatics analyses conducted here highlight the most enduring transfer events from marine to gut *Bacteroidetes*. Phylogenetic trees do not provide any information on the dynamics of gene transfer, particularly on the duration of residence of a newly acquired gene by the host strain.

The newly transferred genes can persist in the microbiome only if there are selection pressures on the host bacterium to keep the new genes. The regular presence of algal food sources in the digestive tract is therefore a likely factor that gives a competitive advantage to the gene-receiving bacterium. Obviously, food and its associated bacteria are potential sources of genetic material, but the algal diet represents the main selection pressure that promoted the retention of genes coding for algal polysaccharide-degrading enzymes.

### What are the substrates of the unknown PL17 enzymes

**Figure 4:**
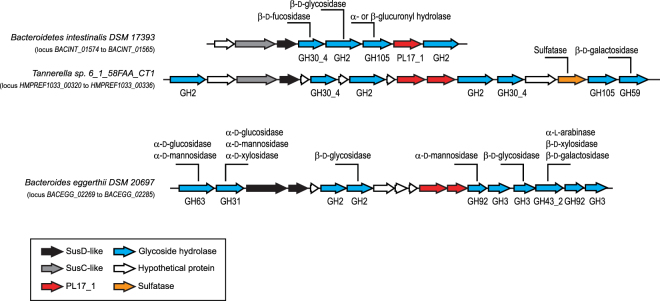
Two types of arrangements in PULs containing PL17 proteins inactive on alginate. These two sets of PULs differed in their organization and, more importantly, in the composition of glycoside hydrolase (GH) family representatives.

The function of several members of family PL17 could not be identified using the collection of polysaccharides that were available to us. To infer what the substrates of these enzymes may be, we examined the nature of the other enzymes encoded in the corresponding PULs. In addition to alginate-utilization loci, PULs of the human gut microbiome containing PL17 genes can be divided into two broad sets according to the composition of the PUL. The first set associates PL17 genes with genes encoding glycoside hydrolases from families GH30_4, GH105 and GH2 (Fig. 4). Family GH105 is composed of α- or β-glucuronyl hydrolases that cleave the Δ-4,5 unsaturated residue at the non-reducing end of the lyase product. Subfamily GH30_4 contains two characterized β-D-fucosidases and family GH2 contains β-galactosidases, β-mannosidases and β-glucuronidases. A literature survey of oligo- and polysaccharides composed of uronic residues, preferably β-D-glucuronic residues, β-D-fucose and β-linked glycosides, identifies, for example, the glycan part of triterpenoid saponins extracted from several plants^37–39^. The corresponding PUL of the strain *Tannerella* sp. 6_1_58FAA_CT B contains a gene coding for a sulfatase in addition to glycoside hydrolases (Fig. 4). Sulfated saponins extracted from marine organisms such as starfish and sea cucumber may also be potential substrates^40,41^.

The PULs in the second set comprise a PL17-encoding gene and several glycoside hydrolases active on α-linked glycosides (GH31, GH63, GH92) and β-linked glycosides (GH2, GH3, GH43_2). This PUL, which contains CAZymes from seven different families, suggests that it targets a very complex polysaccharide whose structure cannot be easily deduced. However, this polysaccharide plausibly has a structure very different from alginate, hyaluronic acid, poly-glucuronic acid and the substrate of the first set of PULs discussed above. The complexity of this putative substrate is probably what prevented us from detecting any activity of several PL17 members using the set of simple substrates at our disposal. Thus, the widely differing genomic environments of PL17-containing PULs and the demonstration that this family is polyspecific indicate that at least two polysaccharide substrates other than those used here are the specific substrates for members of this family.

The clade of the PL17 phylogenetic tree (depicted in green in Fig. 1) that groups together hyaluronan lyase and glucuronan lyase contains a lineage of enzymes found in the human gut microbiome that cluster independently of other enzymes. The deep root of the branch suggests ancient acquisition by the human microbiome. These enzymes did not share the glucuronan lyase activity of the closest characterized enzymes and the protein sequence divergence probably reflects functional adaptation to a substrate yet to be identified. Importantly, although we were not able to attribute a function to these proteins, a BLAST search of their sequences against metagenomics data sets derived from Japanese, Danish, Spanish and American stool samples (GenomeNet Database Resources)^42,43^ revealed the occurrence of these enzymes in populations living on three continents, suggesting a widespread role of these enzymes in the human gut microbiome.

## Conclusion

The diversity of substrate specificity in polysaccharide lyase family PL17 was investigated using a rational bioinformatics selection of enzymes and individual biochemical characterization of recombinant proteins. Two new enzyme activities (hyaluronan and glucuronan lyases) were characterized in the family. The lack of activity on tested substrates for several PL17 members and the analysis of the composition and organization of corresponding PULs suggests at least two other lyase activities in this family.

The phylogenetic analysis of family PL17 suggests the ancient acquisition of alginate lyase genes by the human gut microbiome. These genes probably spread to other bacteria of the microbiome by horizontal transfer between strains and was retained and evolved under selection pressures exerted by diet. This scenario is also likely for the PL6 alginate lyase genes that co-occur with PL17 genes in the alginate utilization loci of *Bacteroidetes* species in the human gut microbiome. The alginate lyase genes were possibly transferred horizontally from a marine *Bacteroidetes*, following a scenario similar to the gene in “porphyran utilization loci”, i.e. via consumption of marine algae. Our analyses suggest that regular consumption of algae acts as a selection pressure on *Bacteroidetes* strains hosting algal polysaccharide-degrading enzymes.

## Methods

### Bioinformatics analysis of polysaccharide lyase family 17

GenBank protein accessions of family PL17 members were extracted from the CAZy database website (www.cazy.org) and used to retrieve the corresponding amino acid sequences from NCBI (www.ncbi.nlm.nih.gov). The amino acid sequences were trimmed to isolate the PL17 catalytic domain based on the three-dimensional structure of *Saccharophagus degradans* (PDB code 4NEI). The resulting sequences were aligned using MUSCLE^44^. The alignment was used to compute a distance matrix using maximum likelihood distances^45^ and BLOSUM62 substitution parameters^46^. The resulting distance matrix was then used to build a phylogenetic tree that was divided into subfamilies using Secator^47^.

### Cloning of alginate lyase genes from family PL17

Thirteen selected genes covering the sequence diversity of PL17 family were cloned as previously described for the PL6 family genes^11^. Genomic DNA and bacterial strains were obtained from the DSMZ-German Collection of Microorganisms and Cell Cultures. The gene names, their accession numbers and the templates (purified genomic DNA or bacterial clones) used for cloning are listed in Table S1. Occurrence of signal peptides and their cleavage sites were identified using SignalP^48^. Primers designed for amplification of the targeted genes without the signal peptide are listed in Table S2. The primers contain sequences of *Nco*I/*Xho*I, *Bam*HI*/Xho*I or *Eco*RI*/Hind*III pairs of restriction enzymes, all of which are compatible for cloning in the expression pET28a plasmid (Novagen, USA).

Given that the genomic DNA of *Y*. *pestis* Z176003, “*Ca*. Solibacter usitatus” and *O*. *bacterium* TAV5 were not available, the genes *yent36911*, *acid4041*, *opit524565* were synthesized and optimized for expression in *E*. *coli*. *yent36911* and *acid4041* were synthesized by MWG Operons (Germany) and cloned in pET28a using *Nco*I and *Xho*I restriction sites at the 5′ and 3′ ends, respectively. *opit524565* was synthesized and cloned in pHTP1 by NZYtech Lda (Portugal).

### Heterologous expression and purification of recombinant PL17 lyases

Protein expression was performed in *E*. *coli* BL21 (DE3) strains induced at OD_600_ = 0.6 with 0.2 mM IPTG for 16 h according to Mathieu *et al*.^11^. Opit_524565 expression was conducted using Rosetta (DE3) pLysS *E*. *coli* strains using auto-inducible media (NZY auto-inducible LB medium – NZTech, Portugal) at 25 °C for 24 h.

Recombinant proteins were purified by affinity chromatography using a nickel agarose affinity resin (Ni-NTA resin, Qiagen) loaded on poly-prep® chromatography columns (Bio-Rad 731-1550) and eluted with buffer 20 mM Tris-HCl pH 8, 500 mM NaCl containing increasing amounts of imidazole (20 mM, 50 mM, 150 mM, 300 mM and 500 mM). After purification, the proteins were desalted on 20 mM Tris-HCl pH 8.3, 150 mM NaCl, 2 mM CaCl2, using a NAP-5 column (GE-Healthcare) as recommended by the manufacturer.

For Acid4041 and Opit527565 proteins, soluble proteins obtained after cell lysis were purified by affinity chromatography using a 1 mL HisTrap™ HP column (GE Healthcare) connected to a NGC chromatography system (Bio-Rad). After a wash step of 5 volumes of buffer A, the protein of interest was eluted with a 20–500 mM gradient of imidazole for 20 min and injected on a ENRich650 column (Bio-Rad), pre-equilibrated with buffer B, 20 mM Tris-HCl pH 7.5, 100 mM NaCl. The purity of the fractions was verified by 10% SDS-PAGE analysis.

### Polysaccharide substrates and activity screening

High molecular weight mannuronan (poly-M) (fraction of guluronic acid F_G_ = 0, [η] = 940 mL.g^−1^) was produced by an *algG*^−^ mutant strain of *Pseudomonas fluorescens*^29^. Alginate with a strictly alternating poly-MG sequence (F_G_ = 0.46 and F_GG_ = 0) was prepared by incubating poly-M with a recombinant mannuronan-C-5 epimerase AlgE4 from *Azotobacter vinelandii* expressed in *Hansenula polymorpha*^30^. Poly-G (F_G_ = 0.95, DP_n_ = 20) was prepared from *Laminaria hyperborea*^28^.

Xanthan and heparin sodium salt were purchased from Dextra Laboratories Ltd. (Reading, United Kingdom). Hyaluronan, dermatan sulfate and gellan were purchased from Glycomix Ltd (Berkshire, United Kingdom). Citrus pectins with 30% and 70% methylation were kindly provided by Cargill (Baupt, France). Sulfated chondroitin was purchased from TCI Europe N.V. (Zwijndrecht, Belgium). Heparan sulfate was a kind gift from Dr. Romain Vivès (IBS, Grenoble, France). Ulvan and alginate from *Ascophyllum* sp. were prepared by CEVA (Pleubian, France). Heparosan and unsulfated chondroitin were prepared Dr. Bernard Priem (CERMAV, Grenoble, France) using genetically modified *E*. *coli* strains^49^.

Screening was conducted according to a protocol adapted from Fer *et al*.^50^. The polysaccharide solutions (125 µL, 0.4% w/v in water) were dispensed into the wells of 96-well filter microplates (10 kDa, PES, Pall) with an equal volume of buffer and 10 µL of desalted enzymes (concentrations ranging from 0.5 µM to 10.6 µM containing 2 mM CaCl_2_). Three pH levels were tested: pH 5 (0.2 M sodium acetate buffer), pH 7 (0.2 M MOPS buffer) and pH 9.2 (0.2 M glycine buffer). A negative control was performed using the buffer without enzyme. Microplates were incubated for 20 h at 25 °C with shaking. After filtration, 80 µL of filtrate was transferred to a microplate (Dutscher) and 160 µL of ferricyanide solution (1.5 g K_3_[Fe(CN)_6_], 24 g Na_2_CO_3_, 1 mL NaOH 5 M, qsp 1 L) were added. The microplate was then heated at 95 °C for 15 min in a thermocycler and cooled to room temperature. The absorbance of 200 µL of sample was read at 415 nm with a microplate reader (Bio-Rad, model 680).

### Degradation kinetics

Enzymatic assays were carried out by incubating 1.4 mL of poly-M, poly-MG and poly-G (0.2% w/v in 50 mM Tris-HCl pH 8, 1 mM CaCl_2_) with a few microliters, ranging from 15 to 50 µL, of the purified enzymes at 25 °C. The protein concentration of the purified enzyme ranged from 5 to 70 µM. The production of reducing ends was measured using the ferricyanide method. Aliquots (100 µL) were transferred to a 900 µL ferricyanide solution (0.3 g.L^−1^ K_3_[Fe(CN)_6_], 24 g Na_2_CO_3_, 1 mL 5 M NaOH, qsp 1 L) which stopped the enzymatic reaction. The solution was heated to 100 °C for 10 min and, after cooling to room temperature, the absorbance of sample was measured at 420 nm with a CARY50 Bio UV/Vis spectrophotometer (Varian).

### Biochemical characterization of Acid4041 and Opit_527565

Salt concentration, pH and temperature optima of Acid4041 were measured using 0.1% of hyaluronic acid and 1 mM CaCl_2_. Initial velocity of polysaccharide degradation was measured at pH levels ranging from 5 to 9 using four 100 mM MES, MOPS and Tris buffers. Optimal temperature was determined at the optimum pH (100 mM MES pH 6.5) at temperatures ranging from 10 °C to 70 °C, the enzyme being pre-incubated for 15 min at the tested temperature. Nine salt concentrations were also tested, from 0 to 400 mM NaCl, at the optimum pH. The biochemical parameters of Opit_527565 were determined according similar protocol. Salt concentration and optimum temperature were measured at in 100 mM Tris buffer pH 7.5 corresponding to the optimum pH (Figs S3 and S4).

### Size-exclusion chromatography

Enzymatic reactions were stopped by heating the samples to 100 °C for 10 min. After filtration (0.2 µm), the samples were eluted on 0.1 M NaCl pre-equilibrated Superdex S200 10/300 and Superdex peptide 10/300 (GE Healthcare) columns mounted in series and connected on an HPLC Ultimate 3000 chromatography system (Thermofischer). The injection volume was 20 µL and the elution was performed at 0.4 mL.min^−1^ in the same eluent. Oligosaccharides were detected by differential refractometry (detector iota 2 – precision instruments) and UV spectrometry at 235 nm (Thermofischer). Retention time of the monosaccharide end-product (4-deoxy-Lerythro-5-hexoseulose uronic acid) was previously determined in Mathieu *et al*.^11^.

### Purification of Acid4041 end products

First, 60 mg of hyaluronic acid was dissolved in 50 mM MES pH 6.5, 20 mM NaCl at 0.4% and degraded with 20 µL of Acid4041 at 3.2 µM, for 20 h at 25 °C with shaking. Then, after enzyme inactivation by boiling and sample filtration on 0.2 µm, the hyaluronan oligosaccharides were purified on three HiLoad® 26/600 Superdex® 30 pg (GE Healthcare) columns mounted in series and connected to a semi-preparative size-exclusion chromatography system which consisted of a Knauer pump (pump model 100), a refractive detector (iota2 Precision instrument) and a fraction collector (Foxy R1) mounted in series. The elution was conducted at a flow rate of 1.2 mL.min^−1^ at room temperature using 100 mM (NH_4_)_2_CO_3_ as eluent. The fraction containing pure oligosaccharides (DP2 and DP4) was collected and freeze-dried.

### ^1^H NMR

Samples were exchanged twice with D_2_O and were transferred into a 5 mm NMR tube. ^1^H NMR spectra were recorded at 323 K using an Advance III 400 MHz spectrometer (Bruker). Chemical shifts are expressed in ppm in reference to water. No suppression of the HOD signal was performed.

## Electronic supplementary material

Supplementary information
