# Supplementary material for: Ancient acquisition of “alginate utilization loci” by human gut microbiota
Source: Sci Rep. 2018 May 23;8:8075. doi: 10.1038/s41598-018-26104-1 (PMC5966431; doi:10.1038/s41598-018-26104-1)
Supplement: Supplementary file 1 — Supplementary information [file 41598_2018_26104_MOESM1_ESM.pdf]

Ancient acquisition of “alginate utilization loci” by human gut microbiota

Sophie Mathieu[1], Mélanie Touvrey-Loiodice[1], Laurent Poulet[1], Sophie Drouillard[1], Renaud Vincentelli[2,3], Bernard Henrissat[2,3,4], Gudmund Skjåk-Bræk[5], William Helbert[1*]

[1] CERMAV, CNRS and Grenoble Alpes Université, BP53, 38000 Grenoble Cedex 9, France

[2] Centre National de la Recherche Scientifique (CNRS), UMR7257, Université Aix-Marseille, Marseille, 13288, France

[3] INRA, USC 1408 AFMB, 13288 Marseille, France

[4] Department of Biological Sciences, King Abdulaziz University, Jeddah, Saudi Arabia

[5] Department of Biotechnology, Norwegian University of Science and Technology, NTNU Sem Sælands vei 6-8, 7491 Trondheim, Norway

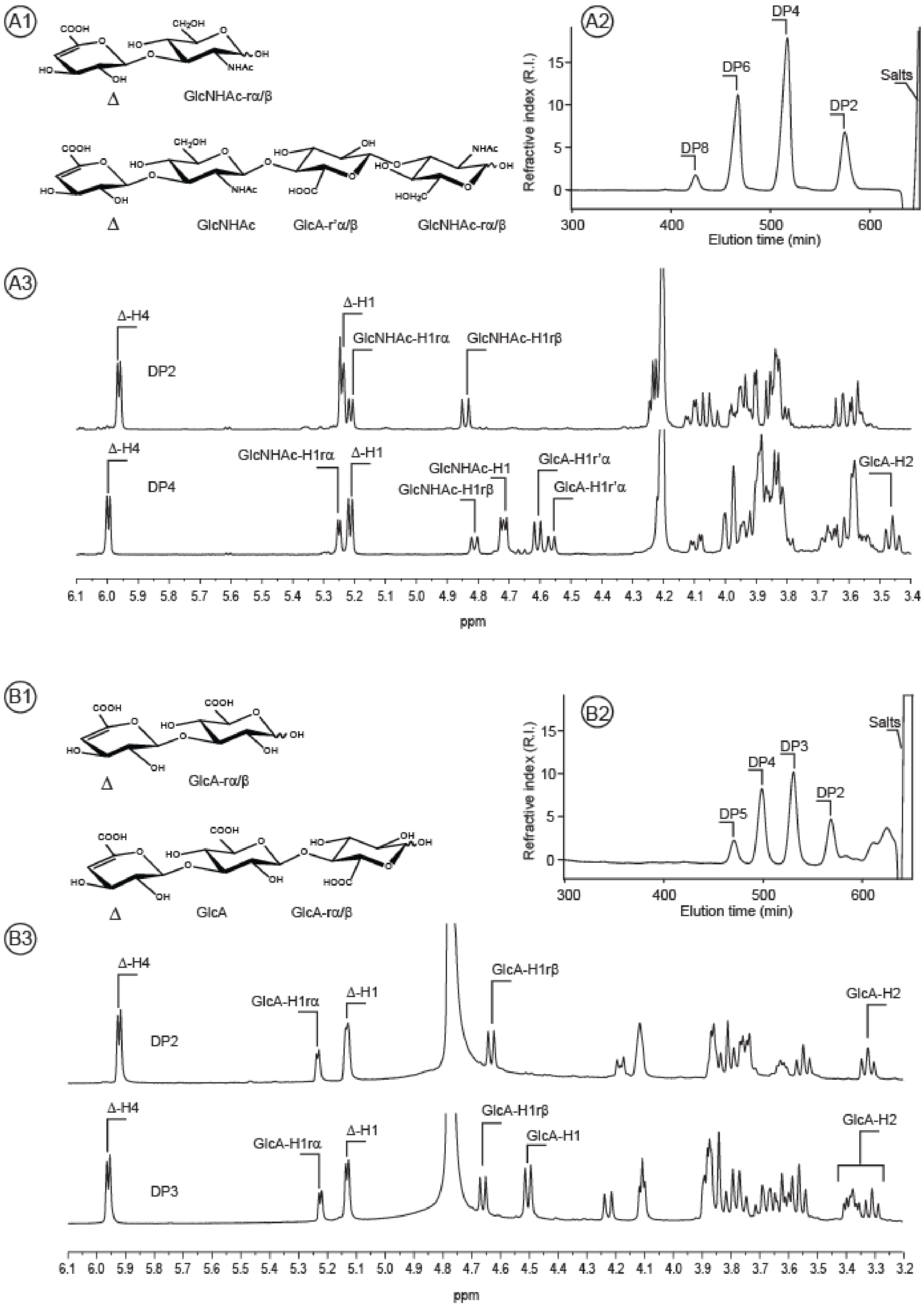

Figure S1: Enzymatic degradation of hyaluronan and glucuronan by enzymes grouped in the PL17_1 subfamily. A: Characterization of the degradation products ogf hyaluranon by the hyaluronan lyases Acid4041 of *Candidatus solibacter usitatus*. A1: Chemical structure of the end-products. A2: Size exclusion chromatography revealing the endo-character of the enzymes. A3: Annotated ^{1}H NMR of the end-product of the hyaluronan lyase. B: Same experiments were conducted with the glucuronan lyase OPIT5_27565 of *Opitutacaea bacterium* TAV5. Structure of the end-products, size exclusion chromatogram and annotated 1H NMR are presented in B1, B2 a,d B3, respectively.

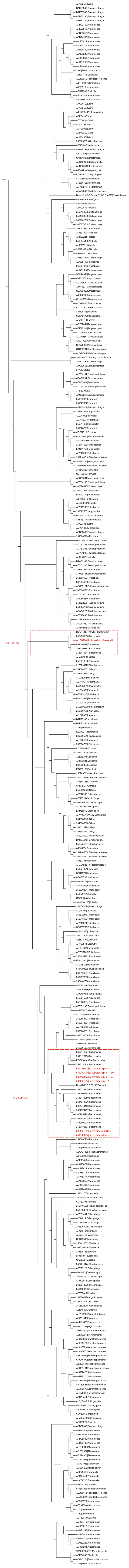

Figure S2 : Phylogenetic tree of the polysaccharide lyases PL6 family. The sequences coding for the alginate lyases found in the human gut microbiote are highlighted.

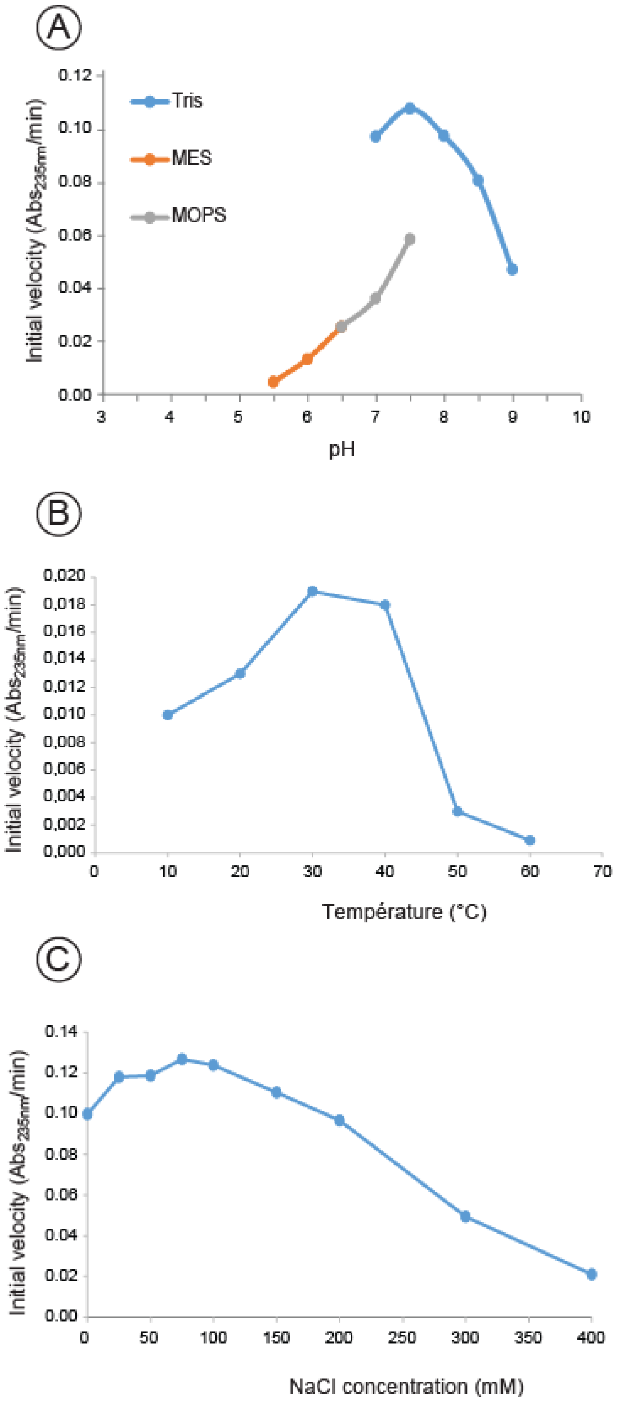

Figure S3: Biochemical characterization of the glucuronan lyase Opit_527565. Determination of pH optimum (**A**), temperature optimum (**B**) and effect of ionic strength on the enzymes activity (**C**).

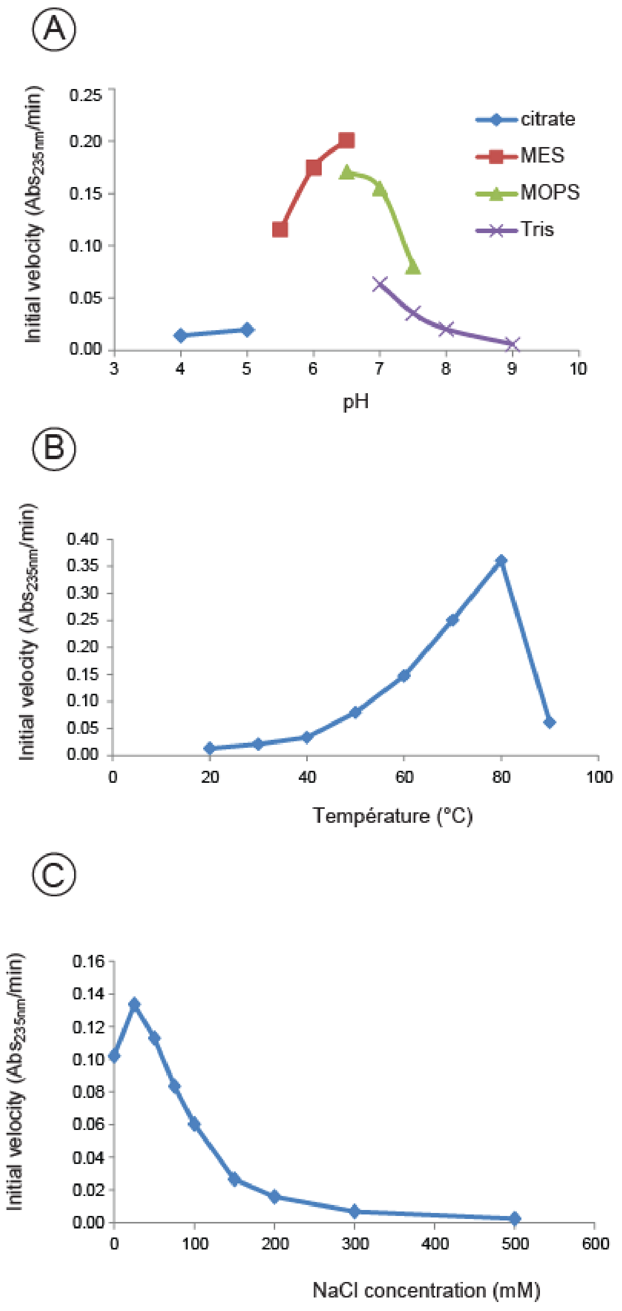

Figure S4: Biochemical characterization of the hyaluronan lyase Acid4041. Determination of pH optimum (**A**), temperature optimum (**B**) and effect of ionic strength on the enzymes activity (**C**).

Table S1. Strains and genes investigated in the study

Strain	Reference	Template used for cloning	Genes	Accession number
Alteromonas macleodii	DSM6062	genomic DNA	Mase04140	AFS36377
Bacteroides eggerthii	DSM20697	genomic DNA	Bacegg03249	WP_004291737
Bacteroides intestinalis	DSM17393	genomic DNA	Bacint01566	ZP_03014006
Candidatus Solibacter usitatus		synthetic gene	Acid4041	ABJ85006
Nonlabens ulvanivorans	DSM22727	bacteria	Nonul2377	WP_036584315
Pedobacter saltans	DSM12145	genomic DNA	Pedsa1196	ADY51764
Pseudoalteromonas atlantica T6c	DSM6840	bacteria	Patl3651	ABG42153
Saccharophagus degradans	DSM17024	genomic DNA	Alg17c	ABD82539
Vibrio alginolyticus	DSM2171	genomic DNA	Valg4459	AGV20268
			Valg4460	AGV20269
Yersinia enterocolitica subsp. *palearctica* Y11	DSM13030	genomic DNA	Yent36911	CBY28839
Yersinia pestis Z176003		synthetic gene	Ypes3069	ADE65978
Opitutaceae bacterium TAV5		synthetic gene	OPIT5_27565	AHF93408.1

Table S2. Primers used for cloning the genes coding for PL17 enzymes

Restriction sites	Gene	Primers (5' → 3')	position of His_6Tag	MW (kDa)
*Nco*I/*Xho*I	Pedsa1196-F	CCA**CCATGG**GACAGCATCCTAATATTATGCTTACC	C-ter	79.5
	Pedsa1196-R	CG**CTCGAG**ATTACCATTTTCAACCAGTTCTGTAAATCGC		
*Nco*I/*Xho*I	Mase04140-F	CGA**CCATGG**GACCTAACTTGGTGATCACGC	C-ter	80.1
	Mase04140-R	CA**CTCGAG**CAGCGCATACACAGCGAAAC		
*Nco*I/*Xho*I	Valg4459-F	CAG**CCATGG**GAACTACACAACCGATTTTGTTGAC	C-ter	82.6
	Valg4459-R	C**CTCGAG**TTTCTCCTGCCCGAATGC		
*Nco*I/*Xho*I	Bacint01566-F	CAG**CCATGG**GATATTTCTTCACTGAAAGCGAC	C-ter	71.2
	Bacint01566-R	C**CTCGAG**AAATTTCCATTCTGTCTTAATTCTCAGTTTCTTATC		
*Nco*I/*Xho*I	Yent36911-F	GGA**CCATGG**GAAAGCAGTTTACGGACAGG	C-ter	78.2
	Yent36911-R	G**CTCGAG**ACTGAATAGTGGTAAGCTACTGTTGG		
*Nco*I/*Xho*I	Bacegg03249-F	CTC**CCATGG**AGCATCCCTGCTTATTGCTTACTC	C-ter	81.6
	Bacegg03249-R	C**CTCGAG**TTTTATTTCGCAGACTCCTTGCCAGC		
*Bam*HI/*Xho*I	Alg17c-F	CGA**GGATCC**GGTCATCCGAATCTTATTGTTACAGAGC	N-ter	82.2
	Alg17c-R	CG**CTCGAG**TTATTTACGTCGAACCACCACGC		
*Bam*HI/*Xho*I	Valg4460-F	CAT**GGATCC**AGCTACCAAACCCAGTCTTAC	N-ter	84.7
	Valg4460-R	C**CTCGAG**TTAAATTTGTGCGAATGCGCC		
*Bam*HI/*Xho*I	Patl3651-F	ACC**GGATCC**GAGCATCCAAATCTGATTATGACC	N-ter	84.5
	Patl3651-R	G**CTCGAG**TTAAGACCCTGCATAGAAATAGG		
*Eco*RI/*Hind*III	Nonul2377-F	GGT**GAATTC**CAAACAGCGATAGAATCTTCTGG	N-ter	84.4
	Nonul2377-R	C**AAGCTT**TTATAATTTAATTAATTGATGTGGTCCATCCCAAC		
